# Differential expression of acetylcholinesterase 1 in response to various stress factors in honey bee workers

**DOI:** 10.1038/s41598-019-46842-0

**Published:** 2019-07-17

**Authors:** Sanghyeon Kim, Kyungmun Kim, Jae Ho Lee, Seung Hee Han, Si Hyeock Lee

**Affiliations:** 1 Department of Agricultural Biotechnology, College of Agriculture and Life Science, Seoul National University, Seoul, Korea; 2 Research Institute for Agriculture and Life Sciences, Seoul National University, Seoul, Korea

**Keywords:** Stress signalling, Entomology

## Abstract

The honey bee acetylcholinesterase 1 (AmAChE1) has been suggested to be related to stress response as judged from its elevated expression level under brood rearing-suppressed conditions. To further investigate the involvement of AmAChE1 expression in the stress response and its physiological functions, we analyzed altered expression profiles of AmAChE1 induced by diverse stress factors. In addition, transcription profiles of several heat shock protein (Hsp) genes (*hsps*) and the vitellogenin (Vg) gene (*vg*) known as general stress markers were investigated as positive references. Among the tested stress conditions, AmAChE1 expression was induced under the brood rearing-suppressed, crowding and heat shock conditions. The *hsps*, particularly *hsp70* and *hsp90*, responded to seven of nine stress conditions tested, confirming that *hsp* expression profiles can serve as a general stress marker. Taken together, AmAChE1 expression is not suitable for using as a stress marker due to its limited response. Nevertheless, AmAChE1 expression appears to be connected, at least in part, to heat shock response and other pathways. Considering that AmAChE1 likely regulates the ACh titer particularly in non-neuronal tissues, thereby modulating the signal cascades mediated by mAChR, the AmAChE1 expression profile under different conditions likely provides important information on its physiological roles in honey bees.

## Introduction

The western honey bee, *Apis mellifera*, plays a major role in the pollination of commercial crops and native plants^1^. In addition to the pollination value, honey bees produce various beneficial products, such as honey, wax, pollen, propolis, royal jelly and venom. The total economic value of honey bees for agriculture in the United States was estimated to be $21.22 to $54.75 billion per year in 2015^2^. Since first reported in the United States in late 2006, colony collapse disorder (CCD), which refers to the phenomenon of sharp declines of honey bee colonies, has been reported in most parts of the world^3^, causing serious damage to the agricultural industry^4^. Although the exact mechanisms of CCD are poorly understood, chronic accumulation of several stressors, including pesticides, parasites, malnutrition and pathogens, is known to cause CCD^5–7^. Therefore, it is imperative to understand the stress response systems of honey bees, and any markers for prior detection of stress in honey bee colonies would be useful for proactive management.

To assess honey bee stress levels, several indicators for stress responses, including physiological, behavioral and cellular stress responses, have been used under various stress conditions^8–10^. In particular, some cellular stress responses such as the expression levels of heat shock proteins (Hsps) and cortico-releasing hormone-binding protein (CRH-BP) have been more recently used to evaluate stress in honey bees under a variety of stressors, including capture, transport, harnessing, cold, heat, and UV light^11^. As various stresses are known to trigger the heat shock response (HSR), Hsps involved in the HSR can serve as stress markers for detecting and quantifying cellular stress levels^12^. Vitellogenin (Vg) has also been suggested as a plausible candidate for a stress marker in that it provides a protective role against oxidative stress and is regulated by the juvenile hormone, which is also considered to be related with stress responses^13,14^. Biogenic amines, such as dopamine, serotonin and octopamine, have also been studied in connection with stress responses^15,16^.

In recent studies, the expression of honey bee acetylcholinesterase 1 (AmAChE1) was determined to be highly correlated with the state of brood rearing by increasing when brood rearing was suppressed^17^. Based on the assumption that the suppression of brood rearing can cause a stressful condition in honey bee colonies, AmAChE1 was suggested to be involved in the stress response or stress management in honey bees^17^. The readthrough AChE (AChE-R), which is a soluble variant of mammalian AChE and likely a molecular homolog of AmAChE1, is induced by various stressors and thus functions as a stress signal molecule^18–20^. In addition, it was reported that the pinewood nematode (*Bursaphelenchus xylophilus*) and fruit fly (*Drosophila melanogaster*) generate soluble AChE upon chemical stress^21,22^. Taken together, these support the notion that AmAChE1 is likely an element of the stress regulation pathway in honey bees.

In this study, to further investigate the involvement of AmAChE1 expression in the stress response and its physiological functions, we analyzed altered expression profiles of AmAChE1 induced by diverse factors, including chemicals (fluvalinate and neonicotinoid), temperature (heat and cold shock), UV-B irradiation, crowding, dehydration, starvation, *Varroa* mite infestation, bacterial challenge (*Escherichia coli* and *Staphylococcus aureus*) and brood-rearing suppression. In addition, we determined the transcription profiles of Hsps (Hsp 10, 60, 70 and 90) and Vg and evaluated the connection between AmAChE1 expression and the Hsp-mediated stress response pathway. The putative roles of AmAChE1 in stress regulation and other physiologies were further discussed.

## Results

### Stresses that induce AmAChE1 expression

Worker bees were treated with each stress factor and then the protein expression levels of AmAChE1 were primarily evaluated and compared with those of control bees, along with transcription levels of AmAChE1 gene (*Amace1*) and several stress marker genes. However, the expression level of AmAChE1 as judged by Western blotting was not well correlated with the mRNA level of *Amace1*. When this discrepancy occurred, the protein expression level of AmAChE1 was primarily used for data interpretation. The results are summarized in Table 1.

**Table 1:** Summarized profiles of AmAChE1 expression and stress marker gene transcription following stresses.

Types of stress			Transcription												Protein expression	
			*hsp10*		*hsp60*		*hsp70*		*hsp90*		*vg*		*ace1*		AChE1	
			H^a^	A^a^	H	A	H	A	H	A	H	A	H	A	H	A
Biotic stress	Bacterial challenge	*E*. *coli*	n^b^	n	n	n	n	n	n	n	n	n	n	n	—^c^	—
		*S*. *aureus*	n	n	n	n	n	n	n	n	n	n	n	n	—	—
	*Varroa* mite infestation		n	n	n	n	n	n	n	n	n	n	n	n	—	—
	Crowding		— ^c^	—	—	—	—	—	—	—	↓	↓	—	↓	↑	↑↑
	Brood rearing suppression	Nurse	—	—	—	—	↑	↑	—	↑↑	—	↑↑	—	—	↑↑↑	↑↑↑
		Forager	↓	—	↓	—	↓	—	—	—	—	—	↓	—	↑↑↑	↑↑↑
Abiotic stress	Starvation		↓	—	↓	—	↓	—	↓	↓	—	—	↑	—	—	—
	Dehydration		—	—	↑	—	↑	—	↑	—	—	—	↑	—	—	—
	Heat shock		↑↑↑	↑	↑↑↑	↑	↑	—	↑↑	—	↑	—	—	—	↑	↑↑↑
	Cold shock		↓↓↓	—	↓↓↓	—	↓↓	↓↓	↓↓	↓	↓	—	↓↓↓	↓	—	—
	Chemical treatment	Imidacloprid	—	—	—	—	—	—	—	—	—	—	—	—	—	—
		Fluvalinate	—	—	—	—	—	——	—	—	—	—	—	—	—	—
	UV irradiation		—	—	—	—	↑↑↑	—	↑	↑↑	—	—	—	—	—	—

Differential transcription levels of stress-marker genes following stresses are marked with symbols of ↑, ↓ (*p* < 0.05), ↑↑, ↓↓ (*p* < 0.01) and ↑↑↑, ↓↓↓ (*p* < 0.001), in which the upward (↑) and downward (↓) arrows indicate overtranscription and undertranscription, respectively, compared to the control. The degree of AmAChE1 expression is marked with ↑, ↑↑ and ↑↑↑ depending on band intensity following Western blotting.

^a^The letters H and A represent the head and abdomen samples, respectively.

^b^The letter ‘n’ indicates no investigation.

^c^The symbol ‘—’ indicates that no difference.

in either protein expression or transcription was observed.

#### Brood rearing suppression

**Figure 1:**
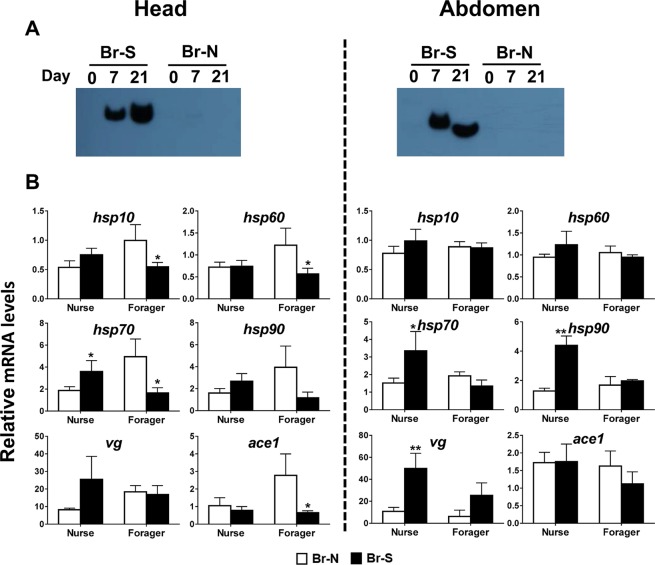
AmAChE1 expression profile and qPCR results of stress marker genes in the head (**A**) and abdomen (**B**) of honey bees following brood rearing suppression. After marking newly emerged honey bees, marked honey bees were collected on day 0, 7 and 21 from three individual hives. Then, brood rearing was suppressed through caging queens and honey bees were collected in the same manner. AmAChE1 expression was detected by Western blotting and qPCR was done with three different biological replications. Br-S is brood rearing suppression condition and Br-N is normal brood rearing condition. The data was analyzed by Student’s t-test, and significant differences were marked with ^*^(*p* < 0.05), ^**^(*p* < 0.01) and ^***^(*p* < 0.001).

AmAChE1 protein expression levels in the worker bees collected on day 0, 7 (nurse) and 21 (forager) from the brood rearing-suppressed and control groups were investigated. On day 7 and 21, significant induction of AmAChE1 expression was observed in both the head and abdomen of brood rearing-suppressed samples whereas no induction was observed in control samples (Fig. 1A), which is consistent with our previous report^17^. In the quantitative real-time PCR (qPCR) results, the stress marker genes showed different expression patterns depending on tissues and age (Fig. 1B). When abdomens of nurse bees were compared, treated groups showed higher transcription levels of *hsp70* (*p* = 0.049), 90 (*p* = 0.001) and *vg* (*p* = 0.009), but this was not observed in the abdomens of forager bees. When forager heads were compared, however, expression levels of *hsp10* (*p* = 0.049), 60 (*p* = 0.051) and 70 (*p* = 0.027) were higher in the control groups compared to the treated groups, whereas no significant differences in abdomens were observed. In forager bees, the overall transcription levels of the *hsp* and *vg* genes were higher in the control compared to nurse bees.

#### Crowding

**Figure 2:**
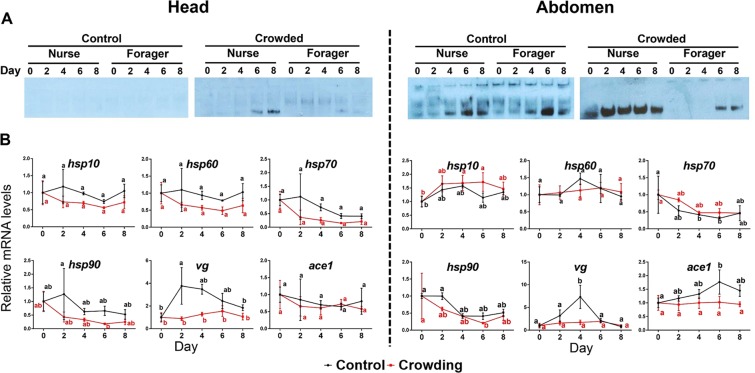
AmAChE1 expression profile and qPCR results of stress marker genes in the head (**A**) and abdomen (**B**) of honey bees following crowding. Two different colonies with similar densities were selected and the density of one was doubled by transferring honey bees from a super into the bottom hive. Honey bees were collected on day 0, 2, 4 and 8. The data was analyzed by two-way ANOVA followed by Tukey’s multiple comparison, and significant differences were marked with ^*^(*p* < 0.05), ^**^(*p* < 0.01) and ^***^(*p* < 0.001).

In the crowding treatment, honey bee workers in the crowded hive showed an increase in AmAChE1 expression, which was more distinct in nurse bees than in forager bees (Fig. 2A). However, expression levels of all the marker genes in the crowding group were not higher than those of the control group on day 4 or 6 in the head samples (Fig. 2B). In contrast, expression levels of *vg* in the head on day 2 (*p* < 0.001) and in the abdomen on day 4 (p < 0.0001) were lower in treated groups than those of the control.

#### Heat shock

**Figure 3:**
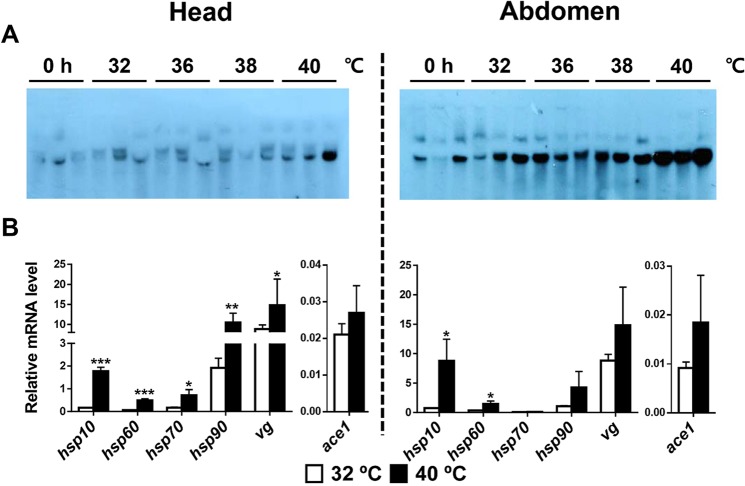
AmAChE1 expression profile and qPCR results of stress marker genes in in the head (**A**) and abdomen (**B**) of honey bees with heat shock treatment. Nurse bees were incubated at 32, 36, 38 and 40 °C for 24 h. Samples incubated at 32, 36, 38 and 40 °C were used to show the AmAChE1 expression level following heat shock (3 replicates per temperature condition) by Western blotting and samples incubated at 32 and 40 °C (3 replicates per temperature condition) were used for qPCR. The data was analyzed by Multiple t-test, and significant differences were marked with ^*^(*p* < 0.05), ^**^(*p* < 0.01) and ^***^(*p* < 0.001).

The AmAChE1 expression was clearly induced by heat shock, which was proportional to the temperature and more apparent in the abdomen than in the head (Fig. 3A). The expression of all the *hsp* and *vg* marker genes was clearly upregulated (*p* value of all the genes < 0.05) in the head upon heat shock (Fig. 3B). In the abdomen, however, expression of *hsp10* (*p* = 0.019) and *hsp60* (*p* = 0.015) only increased in heat-shocked samples compared to the control (Fig. 3B).

### Stresses that do not induce AmAChE1 expression but affect hsp or vg transcription

#### Starvation/Dehydration

Nurse bees were tested for the stress of starvation and dehydration as forager bees are too vulnerable to nutrient deprivation, surviving less than 24 h without a food supply^23^. Both starvation and dehydration did not affect the expression of AmAChE1 (Fig. S1). However, stress marker genes showed opposite expression patterns: their expression was higher in the dehydration group, as reported in other insects^24,25^, but lower in the starvation group compared to the control group (Fig. S2). In general, expression levels of marker genes on day 1 were higher than those on day 0 including the control, which seems to be due to caging stress. However, their absolute levels of expression were lower on day 1 and 2 in the starvation group compared to the dehydration group. In *Drosophila*, the energy consumption was higher during dehydration than starvation^26^, which supports higher expression levels of *hsp*s and *vg* in honey bees during dehydration than starvation.

#### Cold shock

Cold shock did not induce the expression of AmAChE1 in both the head and abdomen (Fig. S3A). However, clear transcription reduction in all of *hsp*, *vg* and *Amace1* genes in the head and *hsp70*, *hsp*90 and *Amace1* genes in the abdomen was observed (Fig. S3B).

#### UV irradiation

Forager bees are exposed to a substantial amount of UV during foraging, whereas nurse bees stay inside dark hives, which likely makes nurse bees more susceptible to UV. With this in mind, Nurse bees were chosen for evaluating UV-B stress. AChE1 expression was not induced by UV-B irradiation in both the head and abdomen (Fig. S4A). However, the transcription of *hsp70* and *90* were upregulated in both the head and abdomen of UV-irradiated bees (*p* < 0.05 except *p* = 0.051 in *hsp70* of abdomen, Fig. S4B).

### Stresses that induce neither AmAChE1 expression nor hsp/vg transcription

Imidacloprid, one of the main causative factors for CCD^7^, and fluvalinate, which is widely used for *Varroa* mite control, were treated with the highest sublethal doses, which resulted in around 10% mortality at 24 h post-treatment. Neither imidacloprid nor fluvalinate treatment affected AmAChE1 expression or stress marker gene transcription (Table 1 or Figs S5 and S6).

As for the biological stress, neither bacterial challenge nor *Varroa* mite infestation induced expression of AmAChE1 (Table 1 or Fig. S7).

## Discussion

### Putative roles of AmAChE1 in stress response of honey bees

In an attempt to investigate the putative roles of AmAChE in the stress response, we investigated the protein expression profiles of AmAChE1 and the transcription profiles of *Amace1* and various stress marker genes under diverse stress conditions. Among the 12 stress conditions exposed to honey bee workers, three of them (brood rearing suppression, crowding and heat shock) induced the expression of AmAChE1 (summarized in Table 1).

AmAChE1 is soluble like the mammalian AChE-R and distributed in both neuronal and non-neuronal tissues, including fat body and hemolymph^17,27,28^. The abundant expression of AmAChE1 in diverse non-neuronal tissues suggests its non-neuronal functions. Recent findings of the essential components of the cholinergic system such as the nicotinic acetylcholine receptor (nAChR) and choline acetyltransferase (ChAT) distributed in non-neuronal tissues^29^ support the presence of a functional and non-neuronal cholinergic system regulated by AmAChE1 in honey bees. As AmAChE1 possesses an extremely low catalytic activity toward ACh compared to the neuronal AmAChE2, it appears to function mainly as an ACh-sequestration protein^27^. With this in mind, the functions of AmAChE1 seem to be associated with the non-neuronal cholinergic system mediating chronic stress response via ACh sequestration rather than with the neuronal cholinergic system modulating acute stress response.

In heat shock treatment, the abdomen sample showed a higher AmAChE1 expression than the head sample. Considering that AmAChE1 expressed in the abdomen is mostly derived from the fat body^17^, expressed AmAChE1 is likely secreted from the fat body and circulates via hemolymph. Detection of soluble AChE with weak enzyme activity in honey bee hemolymph supports this notion^28^. Thus, the heat-induced expression of AmAChE1 appears to exert systemic effects on the whole body. Based on the facts that the toxicity of organophosphorus (OP) and carbamate (CB) insecticides increases in proportion to temperature increase^30^ and that AmAChE1 provides chemical defense against OP and CB insecticides^27^, the augmented AmAChE1 level likely provides extra protection against toxic chemicals particularly under heat-stressed conditions. Another possible scenario is that AmAChE1 circulating the body reduces the overall amount of ACh through sequestration or degradation, thereby regulating the cholinergic signal pathways. Thus, ACh concentration reduction in hemolymph by AmAChE1 would selectively influence the physiology of different tissues. In humans, plasma ACh can promote macrophage phagocytosis via stimulation of nAChR, resulting in enhancement of the cellular immune response^31^. Although the same ACh-related immune pathway has not been reported in honey bees, the recent identification of major cholinergic components such as ChAT and nAChR in honey bee hemocytes suggests the existence of functional ACh-mediated immune regulation pathways, especially in non-neuronal tissues^29^, and further indicates the role of AmAChE1 in the immune system of honey bees. If this is the case, AmAChE1 overexpression following heat shock is expected to reduce the ACh concentration, thus resulting in the downregulation of the immune response of individual bees.

Interestingly, transcription of all tested *hsp* genes was induced in bees that received heat shock, suggesting the activation of the HSR pathway (Table 1 and Fig. 3B). Thus, the putative reduction of immune competence appears to be connected with an enhanced HSR. This finding is consistent with the report by McKinstry *et al*. (2017), where heat shock suppressed the immune gene transcription in the abdomen whereas the immune challenge to the abdominal cuticle resulted in decreased expression of multiple HSR genes^32^. Such a mutually antagonistic relationship between the HSR and immune response was explained by the trade-offs theory of ecological immunology^33^. In the eusocial honey bees, as both the HSR and immune response require large energy resources, trade-offs between these two responses at the individual level would be necessary to allocate limited resources in a manner that eventually benefits the colony^32^.

Under the brood rearing suppression condition, which was generated by confining the queen bee in a plastic queen cage, AmAChE1 expression was induced and maintained from at least day 7 to day 21. This result is consistent with the previous finding when brood rearing suppression was induced by confining the entire hive inside a screen tent, which completely blocked the free foraging activity^17^. Taken together, AmAChE1 expression is induced in both nurse and forager bees by brood rearing suppression regardless of forager bee activity.

Although AmAChE1 expression by brood rearing suppression was equally induced in both nurse and forager bees (Fig. 1A), the overall stress level, as judged by the transcription levels of the reference *hsp* and *vg* genes, was more apparent in nurse bees compared to forager bees (Fig. 1B). Under the brood rearing-suppressed condition, the blocking of nurse bee’s blood care activity is expected to directly influence the nurse bee’s physiology, thus resulting in more cellular stress, which is also suggested by a more selectively enhanced HSR in nurse bees. In the case of forager bees under the normal condition, rebuilding of the neural network is required for enhanced learning and memory capacity while foraging outside^34^. As the enhanced proteostasis mediated by the HSR particularly in the head would be necessary to reinforce the neural function of foragers, it appears natural to observe the generally higher transcription levels of *hsp* genes in the control forager bees than in the treated foragers. Nevertheless, the lack of precise correlation between AmAChE1 expression and *hsp* gene transcription suggests that the HSR is not directly connected to the AmAChE1 expression pathway.

Considering that the ACh-mAChR pathway is involved in the regulation of energy metabolism^35^, the expression of AmAChE1 in the abdomen (i.e., fat body) of both nurse and forager bees under the brood rearing-suppressed condition can be speculated to cause the alteration of energy metabolism. In rats, one of the mechanisms regulating secretion of insulin, the main component of energy metabolism, is mediated by the binding of ACh to the M3 mAChR subtype of pancreatic β cells^35,36^. All of these may suggest some possibility of metabolic regulation mediated by the AmAChE1-ACh-mAChR circuit, perhaps via the insulin/insulin-like growth factor signaling (IIS) pathway in honey bees.

Crowding treatment induced the expression of AmAChE1, which was more apparent in nurse bees than in forager bees. This differential expression of AmAChE1 between nurse and forager bees appears to be due to the fact that nurse bees stay inside the hives, thus being constantly exposed to the crowding condition, whereas forager bees spend most of their time outside for foraging during the daytime and are less exposed to the crowding condition. Considering that a bee colony is a superorganism consisted of numerous worker bees with different functions, the increase in colony density may alleviate an individual worker bee’s workload, which likely reduces the cellular stress level in individual bees. Overall downregulation of the *hsp* and *vg* transcription under the crowding condition supports this notion. If this is the case, the crowding condition (i.e., doubling of colony density) may not act as a stress factor to honey bees at least under the experimental conditions used in this study. If assuming that colony-level immunity can be boosted by increased colony density, the putative downregulation of the immune response by overexpressed AmAChE1 in individual worker bees would be compensated.

ACh also might be related with nestmate recognition. Treatment of a muscarinic agonist to young honey bees improves recognition of nestmates whereas muscarinic antagonist treatment increases attacks on nestmates^37^. If assuming that a sudden increase of population could work as a signal for extrinsic invasion to the colony, it can be speculated that the AmAChE1-mediated reduction of the ACh titer leads to a decrease in the sensitivity for nestmate recognition, thereby temporarily enhancing a colony’s defense capability.

### The discrepancy between the levels of AmAChE1 protein and Amace1 mRNA

Interestingly, the levels of AmAChE1 protein were not well correlated with the *Amace1* transcription levels. This inconsistency between mRNA and protein levels suggests that certain post-transcriptional control, such as pumilio-mediated translational regulation, likely plays an important role in the expression of AmAChE1. As the *Amace1* transcript has six pumilio-binding domains in the 3’ UTR and one in the 5’ UTR (Table S2), it can be speculated that the pumilio-mediated mechanism is involved in the regulation of the ultimate expression level of AmAChE1 along with the typical control mechanism for *Amace1* mRNA degradation. Therefore, the lack of correlation between the levels of *Amace1* mRNA and AmAChE1 protein observed under different conditions is likely due to the presence of multiple inter-connected pathways that regulate the expression of AmAChE1 including the control pathways of *Amace1* mRNA degradation and translation.

### Putative roles of HSPs and Vg in stress response of the honey bees

Among the *hsp* genes tested, both *hsp70* and *hsp90* responded broadly to a wide variety of stress factors (brood rearing suppression, starvation, dehydration, heat shock, cold shock and UV-irradiation), suggesting their potential as general stress markers (Table 1). Increased transcription of *hsp70* and h*sp90* upon heat stress was also reported in a previous study^38^. Both Hsp70 and Hsp90 form a multichaperone complex with the Hsp-organizing protein (Hop) and assist the folding of signaling-related proteins such as kinases, steroid hormone receptors and transcription factors^39^. The overtranscription of *hsp70* and *hsp90* particularly in brood rearing-suppressed nurse bees likely suggests an increased rate of protein translation, which is in good agreement with the over-represented protein biosynthesis pathway under the brood rearing-suppressed condition, as observed in the gene-set enrichment analysis of worker bee transcriptomes^34^. Likewise, both *hsp70* and *hsp90* were overtranscribed upon UV-B irradiation stress, indicating that Hsp70 and Hsp90 function as chaperones to protect or rescue UV-damaged proteins in nurse bees. Hsp60, the mitochondrial protein, is induced by diverse stress factors such as water-immersion^40^, metals, heavy metals and chemicals^41–43^. Hsp10, as a co-chaperonin of Hsp60, makes a complex with Hsp60^44^, which explains the similar transcription patterns of *hsp10* and *hsp60*. Although both Hsp10 and Hsp60 were not induced by chemical treatment unlike previous reports^41,43^, high induction by heat shock suggests their important role for protecting cells from stress.

In this study, five among nine stress conditions affected the transcription of *vg* (Table 1). The heat shock and brood rearing suppression induced the overtranscription of *vg*, whereas cold shock, crowding and starvation resulted in reduced expression. The transcription of *vg* did not change in other stress treatments. Vg, the yolk protein of oviparous organisms, is related to oxidative stress, aging, hormonal dynamics, caste differentiation and gustatory responsiveness in honey bees^13,14,45–47^. With such close connections to the various physiological states of honey bees, Vg has been suggested as a molecular stress marker^48^. Similar to our results, an increase of Vg was observed in the heat-shocked and brood rearing-suppressed honey bees^49,50^, whereas a decrease was observed in the *Nosema ceranae*-infected^51^ and acaricides-treated honey bees.

Transcription of *vg* was downregulated under the crowding, starvation and cold shock conditions (Table 1). The reduced expression of Vg, as an antioxidant, seems natural if it is assumed that the reduced metabolic rate is commonly resulted from these conditions due to the following respective factors (i.e., reduced labor, depleted nutrients and reduced temperature under the crowding, starvation and cold shock conditions, respectively)^52^, which results in the reduction of metabolism-related oxidative stress. Under these conditions of *vg* downregulation, all the *hsp* genes tested were also undertranscribed. This simultaneous undertranscription of both *hsp* and *vg* genes following the crowding, starvation and cold shock conditions may suggest that these genes are responding to the oxidative stress level in honey bees.

As reported in the midge *Belgica Antarctica*^53^, in which *hsp70* and *hsp90* transcript levels increased following UV irradiation, we initially expected an increase in *vg* transcription after UV-B irradiation. However, the *vg* transcript level was somewhat decreased in UV-treated groups than control groups although it was not statistically significant (*p* = 0.068 in abdomen). Since an increase of the JH titer and decrease of Vg are the two major factors for nurse-forager transition^13^ and pre-exposure to sunlight increases the JH level of young bees^54^, it can be speculated that the UV irradiation in fact accelerated the nurse-forager transition. The overall reduction in *vg* transcription in nurse bees following UV exposure can be explained if it is assumed that the necessary Vg reduction for the labor-transition following UV irradiation masks the demand for a Vg increase for the UV-induced oxidative stress.

### Crosstalk pathways between AmAChE1 expression and HSR

The AmAChE1 expression was accompanied by the transcription of all tested *hsp* genes following heat shock. Although not as complete as the heat stress, brood rearing suppression also resulted in both AmAChE1 expression and the transcription of two *hsps* (*hsp70* and *hsp90*). This simultaneous upregulation of AmAChE1 and *hsps* under the stress conditions suggests that there is crosstalk between the expression of AmAChE1 and Hsps. Transcription of *hsps* is controlled by heat shock factor 1 (HSF1), of which activation is modulated via various protein kinase pathways by heat shock^55^. Considering that HSF1 is specific for *hsp* transcription, activation of *Amace1* may not be directly regulated by HSR but rather by the upstream kinase pathways. Considering that Hsp expression is regulated by mAChR activation in the rat hippocampus^56^ and AmAChE1 regulates the ACh titer, perhaps by sequestration, AmAChE1 likely exerts its functions via the mAChR-mediated pathway in the honey bee as well. If this is the case, as the cAMP-dependent protein kinase (PKA) pathway is known to activate HSF1^55^, AmAChE1 appears to exert its function via the PKA pathway mediated by mAChR, thereby modulating the stress response. In contrast, the mutually antagonistic relationship between the HSR and the immune response, putatively mediated by AmAChE1, also suggests the presence of antagonistic crosstalk between the HSF1 activation and immune regulation

Under the crowding condition that induced AmAChE1 expression, however, transcription of all *hsp* genes exhibited the opposite tendency. If assuming that overexpression of Hsps reflects various cellular stresses, this finding supports the notion that the crowding condition in fact did not induce cellular stress. The lack of correlation between AmAChE1 expression and *hsp* transcription further suggests that AmAChE1 expression does not necessarily indicate the presence of cellular stress in honey bees.

### Potential of AmAChE1 as a marker for physiological changes

By determining the AmAChE1 expression profile following diverse stress factors, we tried to evaluate the possibility of AmAChE1 as a general stress marker in honey bees. Unlike *hsp* genes, particularly *hsp70* and *hsp90* that responded to six of nine stress conditions tested, AmAChE1 expression was only induced under three out of 12 stress conditions. Thus, these results suggest that AmAChE1 is inappropriate as a general stress marker. However, AmAChE1 expression appears to be connected, at least in part, to the HSR. Considering that AmAChE1 regulates the ACh titer particularly in non-neuronal tissues, thereby modulating the signal cascades mediated by mAChR, the AmAChE1 expression profile in honey bees likely provides important information on physiological changes in honey bees.

## Materials and Methods

### Insects

Colonies of western honey bees (Italian hybrid) have been kept at the Gwanak campus of Seoul National University (Seoul, Korea, 37°27′46.8″N, 126°57′06.9″E). Young worker bees located in the center of brood rearing cells were considered as nurse bees and old worker bees that enter the hive from foraging and are mostly found around the honey/pollen cells were considered as forager bees. Nurse or forager bees were collected from combs with a brush, placed in a plastic cage and briefly anesthetized with CO_2_ gas (30 s). Anesthetized bees were placed in a 474-ml transparent plastic disposable cup (30 workers/cup) and provided with a piece of pollen bread (1 cm3) and 50% sucrose solution in a 5-ml plastic syringe. These bees were maintained at 32 °C in 40–60% relative humidity under a dark condition, which was the standard test condition in this study. The 50% sucrose solution was replaced every day. When exact age synchronization was needed, newly emerged worker bees were marked with paint (Uni posca, Mitsubishi pencil, Japan) and collected later at appropriate dates. After each treatment, honey bees were flash-frozen with liquid nitrogen and stored in a −80 °C deep freezer until used. All stress experiments along with appropriate controls were conducted with three biological replicates.

### Stress treatment

#### Biotic stress

Bacterial challenge (Escherichia coli and Staphylococcus aureus): *E*, *coli* and *S*. *aureus* were cultured in LB broth with gentle shaking until the OD_600_ value became 0.6 and the resulting cultures (0.5, 2.5 and 12.5 ml) were centrifuged at 13,000 rpm for 5 min. Each pellet was mixed with 4 ml of 10% sucrose solution to obtain the final working solution (0.6 × 10^8^, 3.0 × 10^8^ and 1.5 × 10^9^ cells/ml). Nurse bees were oral-challenged by providing with 10% sucrose solution containing bacteria under the standard condition. Control nurse bees were provided with a 10% sucrose solution without bacteria. Both bacteria-challenged and control honey bees were collected after 24 h.

*Varroa* mite infestation: Live *Varroa* mites were collected from a *Varroa* mite-infested beehive using sugar powder^57^. Test nurse bees were collected from a *Varroa* mite-uninfested hive. Two or three *Varroa* mites were manually attached to each of the healthy nurse bees. *Varroa* mite-attached and unattached control nurse bees were incubated respectively under the standard condition for 24 h and collected. Nurse bees naturally infested with *Varroa* mites in the infected hive were also collected as a reference.

Brood rearing suppression stress: For the collection of honey bee workers under the normal brood rearing condition, newly emerged workers were paint-marked from hives actively engaged in brood rearing. The paint-marked bees were then collected on day 0, 7 and 21, flash-frozen and immediately stored at −80 °C until use. To create the brood rearing-suppressed condition, honeycomb containing eggs, brood and pupa were removed from the hive and the queen was confined inside a plastic queen cage with a few nurse bees. On the same day, newly emerged workers were marked with paint and then collected in the same way on day 0, 7 and 21. Collected bees were flash-frozen and immediately stored at −80 °C until use.

#### Abiotic stress

Starvation/Dehydration: For starvation stress, nurse bees were provided with only water instead of a 50% sucrose solution, with other conditions being equal to the standard condition. For dehydration stress, nurse bees were supplemented with solid sucrose instead of a 50% sucrose solution under the same standard condition. Control bees were maintained under the standard condition. Treated and control bees were collected after 24 and 48 h^58^.

Crowding stress: Similar colony densities (approximately 25,000 bees/hive) were maintained in two different hives, each with a super (a total of 16 combs/hive). For the crowding treatment, bees in a super (eight combs) of one hive were transferred into the bottom hive, thereby roughly doubling the density. The other hive was maintained without any change in colony density and used as the control. Nurse bees and forager bees from each hive were collected on day 0, 2, 4, 6 and 8.

Thermal stress (heat and cold shock): For both heat shock and cold shock treatments, nurse bees were used considering that forager bees are normally exposed to a diverse range of temperatures and adapt more efficiently to heat shock through the expression of Hsp^59^. In contrast, nurse bees are less often exposed to low temperatures compared to forager bees, thus being more likely to be vulnerable to cold shock. Nurse bees were collected from a hive and immediately placed in separate incubators with different temperature settings (32, 36, 38 and 40 °C for heat shock and 32, 27, 22 and 17 °C for cold shock). Treated bees were incubated under the standard condition except for the temperature and collected at 24 h post-treatment.

Chemical stress (fluvalinate and neonicotinoid treatment): For the treatment of imidacloprid, of which most natural exposure to honey bees is via the oral route, forager bees were provided with a 50% sucrose solution containing 0, 4, 16, 64, and 256 ppb imidacloprid, with other conditions being equal to the standard. Treated bees were collected after seven days of incubation.

As for the treatment of fluvalinate, of which most plausible exposure is via contact, forager bees were placed in a Mason jar precoated with an appropriate amount of fluvalinate and then maintained for 14 days with the provision of pollen bread and a 50% sucrose solution. The Mason jar was coated with 5 ml fluvalinate-acetone solution (0, 3.2, 16, 80 and 400 ppm) using a rolling machine under a fume hood. Bees were collected 14-days post-treatment.

UV irradiation (oxidative stress): Under the assumption that nurse bees are more sensitive to UV irradiation, paint-marked seven-day-old nurse bees were used for the UV irradiation. Nurse bees were collected from a hive without direct exposure to sunlight. Half of the collected bees were then exposed to UV (peak at 305–310 nm wavelength) generated by a 20 W UV-B lamp (G20T10E, Sankyo Denki., Kanagawa, Japan) at a distance of 15 cm away for 4 h under the standard condition and collected immediately. The other half of the collected bees were placed in the dark for 4 h under the standard condition and collected as the control.

### Native-polyacrylamide gel electrophoresis(PAGE) and Western blotting for AmAChE1 expression confirmation

Honey bee samples were separated into the head and abdomen. The abdomen was further dissected to remove the gut and the gut-removed abdomen carcass was used as the ‘abdomen’ sample in this study. For protein extraction, 10 heads or abdomens were homogenized with 0.1 M Tris-Hcl buffer (pH 7.8) containing 0.5% Triton X-100 and a protease inhibitor cocktail (Merck, Darmstadt, German) using a bullet blender (Next Advance, NY, USA). The homogenized samples were centrifuged at 12,000 g at 4 °C for 10 min. The supernatants were filtered through column type D (GeneAll, Seoul, Korea) to remove lipids. The BCA assay was used to determine protein concentrations of the extracted protein samples.

To determine whether AmAChE1 expression is involved in responses to different stress, protein expression levels of AmAChE1 following treatment of various stressors were primarily evaluated by Western blotting and compared with those of the control bees^60^. An aliquot (10 μg) of a protein sample was separated on a 7.5% native-polyacrylamide gel in duplicate at 120 V for 90 min. After the native-PAGE, Western blotting was performed with anti-insect AChE1 antibody as previously described^17^.

### Quantitative real-time PCR

To measure mRNA levels of genes, qPCR was conducted. Total RNA was extracted from 10 heads or gut-removed abdomens with TRI reagent (MRC, Cincinnati, OH, USA) following the instructions of the manufacturer. The extracted RNA was treated with DNaseI (TAKARA Korea Biomedical Inc., Seoul, Korea) to remove genomic DNA. After DNaseI treatment, the first strand cDNA was synthesized from 2.5 μg of total RNA with Superscript IV reverse transcriptase (Invitrogen, Carlsbad, CA, USA) at 55 °C for 10 min.

The *hsp 10*, *60*, *70 and 90* as representatives of four major insect Hsp families, small Hsps, Hsp60, 70 and 90 families^61^, and the Vg gene (*vg*) were used as reference genes for stress along with the (*Amace1*). PCR primers for the stress reference genes and the ribosomal protein S5 (RPS5) gene, which was used as a qPCR reference gene, are listed in Table. S1. qPCR was conducted with SYBR Premix Ex Taq (TAKARA Korea Biomedical Inc., Seoul, Korea) in 40 cycles of the following thermal program: 5 s at 95 °C, 15 s at 56 °C and 15 s at 72 °C. The transcription level of *Amace1* along with those of the reference stress genes was evaluated following various stress treatments.

### Statistical analysis

A statistical analysis of the qPCR data was performed using Prism 6.0 (GraphPad, San Diego, USA). For a comparison of two-sample data, Student’s t-test was performed and a significant difference was marked with ^*^(*p* < 0.05), ^**^(*p* < 0.01) and ^***^(*p* < 0.001). For comparison of multiple sample data, one-way or two-way ANOVA test followed by Tukey’s multiple comparison test was performed, and significant differences in mean values (*p* < 0.05) were marked with different alphabets.

## Supplementary information

Supplementary file
