# Supplementary material for: Differential expression of acetylcholinesterase 1 in response to various stress factors in honey bee workers
Source: Sci Rep. 2019 Jul 17;9:10342. doi: 10.1038/s41598-019-46842-0 (PMC6637154; doi:10.1038/s41598-019-46842-0)
Supplement: Supplementary file 1 — Supplementary file [file 41598_2019_46842_MOESM1_ESM.pdf]

# Supplementary Information

## Differential expression of acetylcholinesterase 1 in response to various stress factors in honey bee workers

Sang Hyeon Kim[1], Kyung Mun Kim[1], Jae Ho Lee [1], Seung Hee Han[1] and Si Hyeock Lee[1, 2]

[1]Department of Agricultural Biotechnology, College of Agriculture and Life Science, Seoul National University, Seoul Korea
[2]Research Institute for Agriculture and Life Sciences, Seoul National University, Seoul Korea

*Corresponding author, shlee22@snu.ac.kr (SHL)

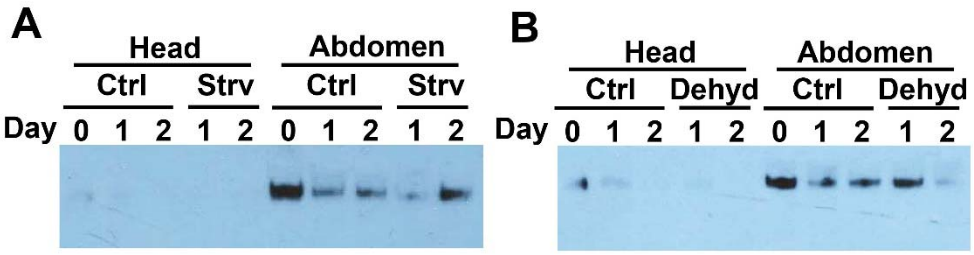


Figure S1. **AmAChE1 expression profile in honey bees under starvation (A) and dehydration (B) conditions.** Nurse bees were incubated for 48 h without sugar or water. Bees were collected at 0, 24 and 48 h.

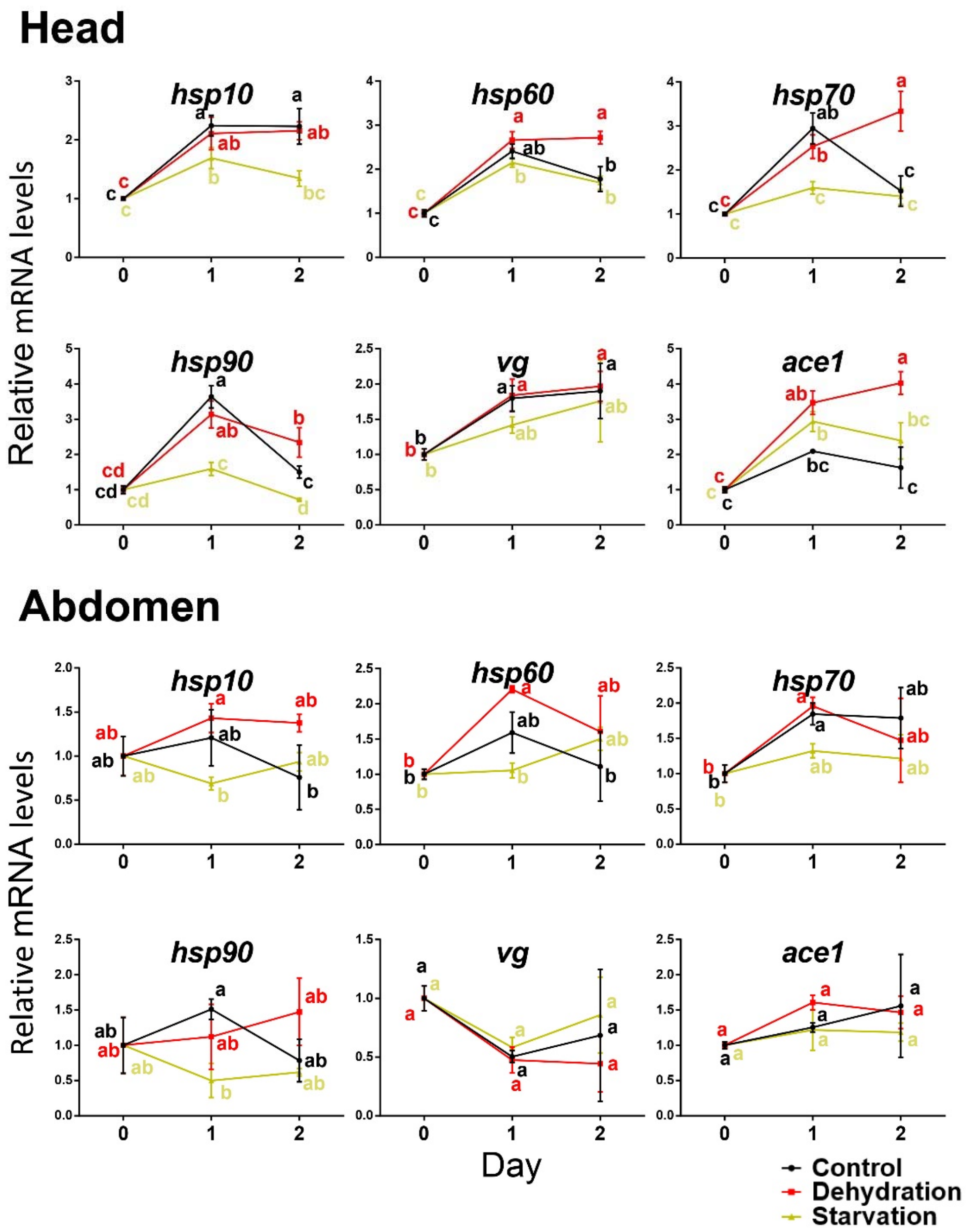


Figure S2. **qPCR results of stress marker genes and *ace1* following starvation and dehydration.** The values of starvation and dehydration were compared with that of the control, and the difference was marked on the graph. The data was analyzed by two-way ANOVA followed by Tukey's multiple comparison and significant differences were marked with * ($p$ < .05), ** ($p$ < .01) and *** ($p$ < .001).

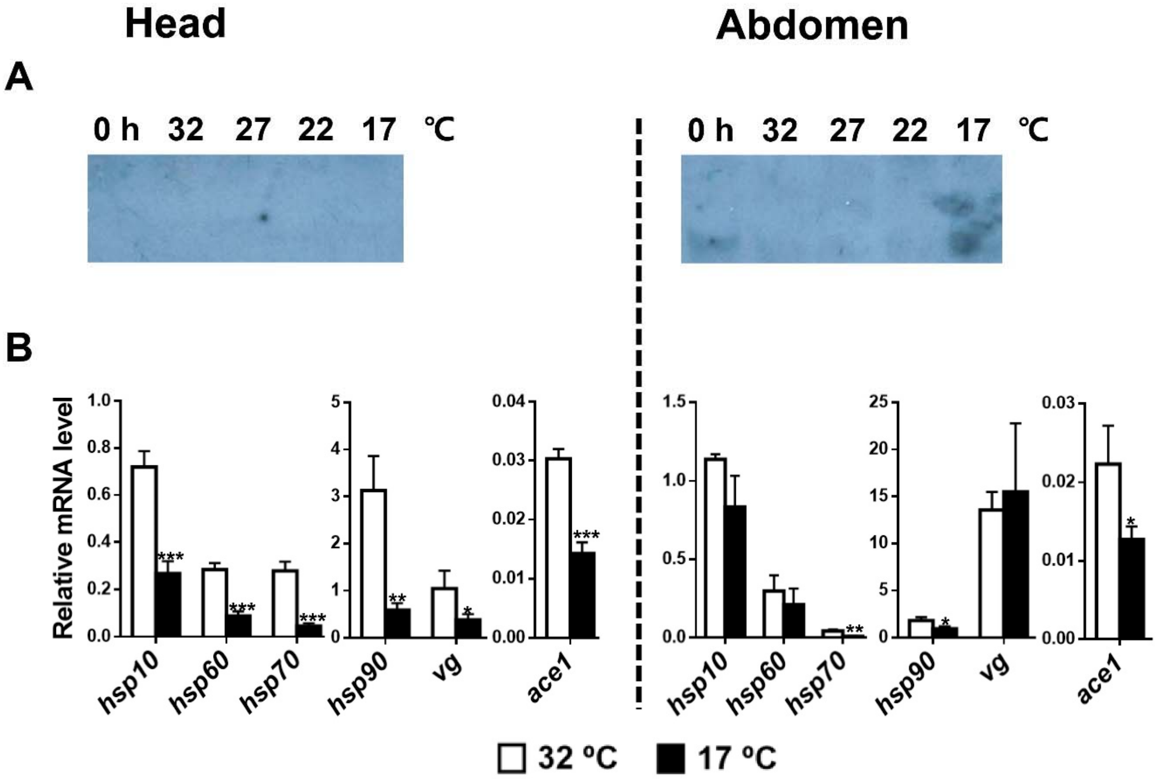


Figure S3. **AmAChE1 expression profile and qPCR results of stress marker genes in the head (A) and abdomen (B) of honey bees with cold shock treatment.** Nurse bees were incubated at 32, 27, 22 and 17ºC for 24 h. Samples incubated at 32, 27, 22 and 17ºC were used to show AmAChE1 expression level following cold shock and samples incubated at 32 and 17ºC were used for qPCR. The data was analyzed by Multiple t-test and significant differences were marked with $^{*}$ ($p < .05$), $^{**}$ ($p < .01$) and $^{***}$ ($p < .001$).

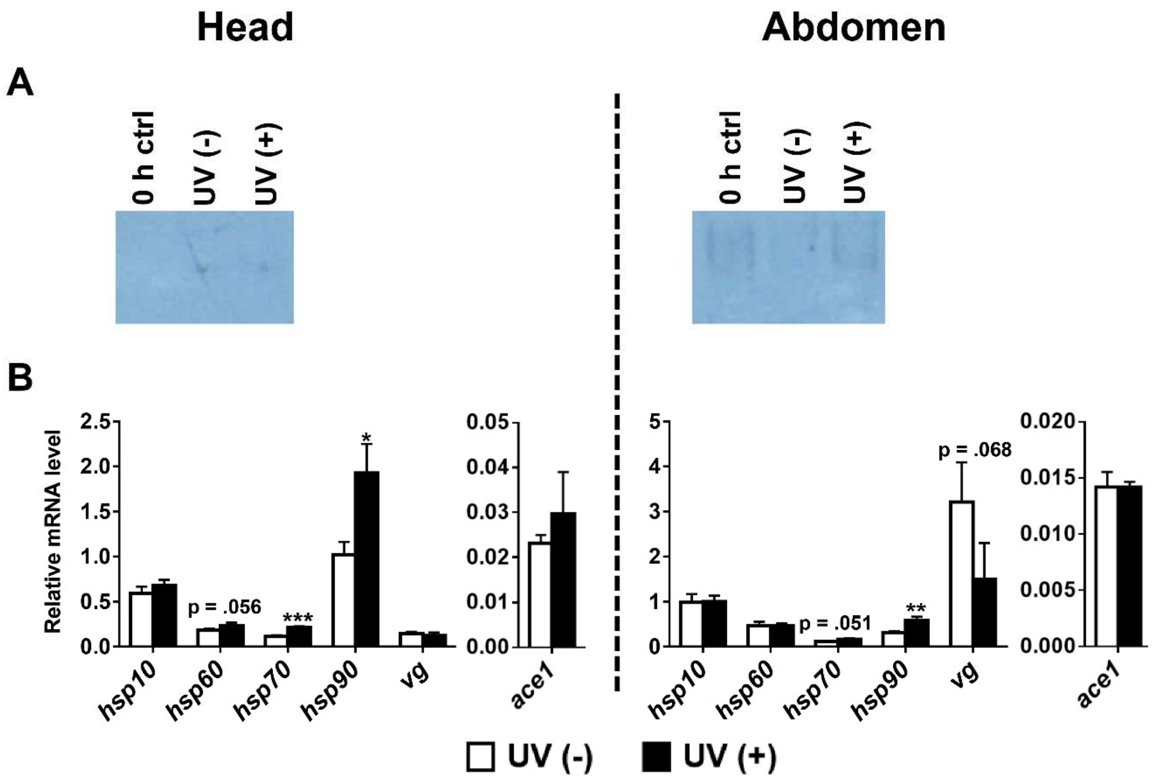


Figure S4**. AmAChE1 expression profile and qPCR results of stress-marker genes in the head (A) and abdomen (B) of honey bees with UV-B treatment.** Seven-day-old nurse bees were exposed to UV-B for 4 h and collected. The data was analyzed by Multiple t-test and significant differences were marked with $^{*}$ ($p < .05$), $^{**}$ ($p < .01$) and $^{***}$ ($p < .001$).

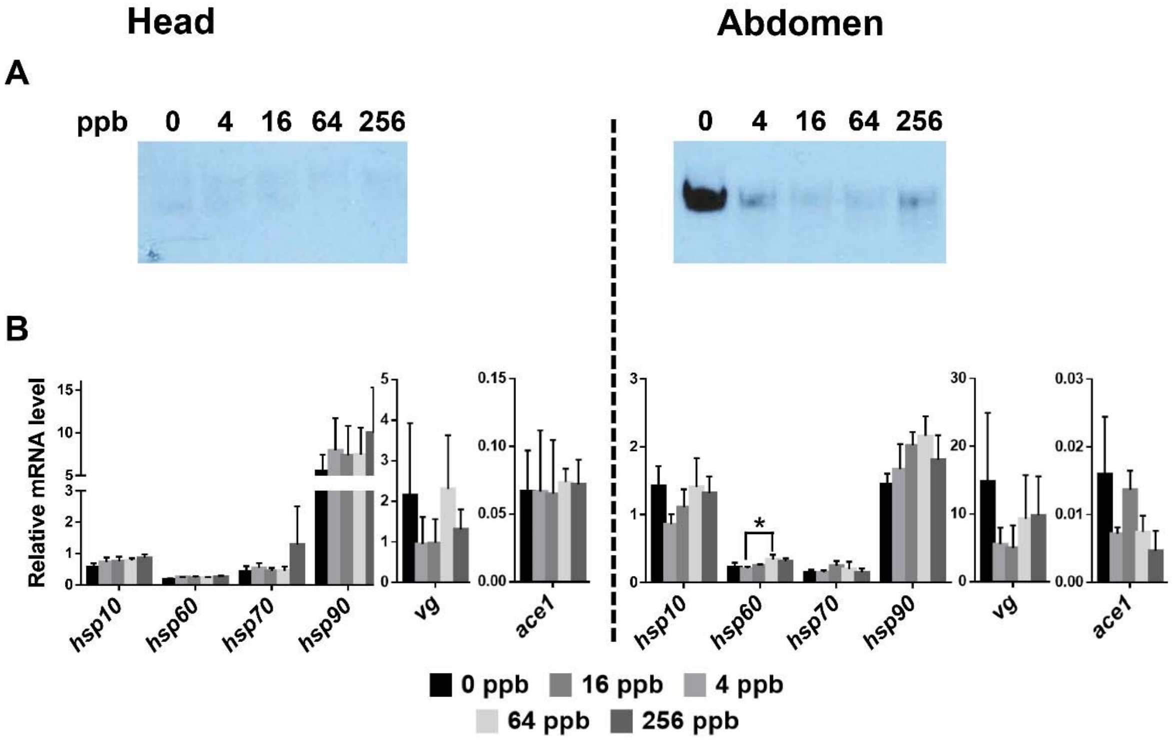


Figure S5. **AmAChE1 expression profile and qPCR results of stress marker genes in the head (A) and abdomen (B) of honey bees with imidacloprid treatment.** Forager bees were incubated while being provided with a 50% sucrose solution containing 0, 4, 16, 64, and 256 ppb imidacloprid for 7 days. The data was analyzed by one-way ANOVA followed by Tukey's multiple comparison and significant differences were marked with * ($p < .05$), ** ($p < .01$) and *** ($p < .001$).

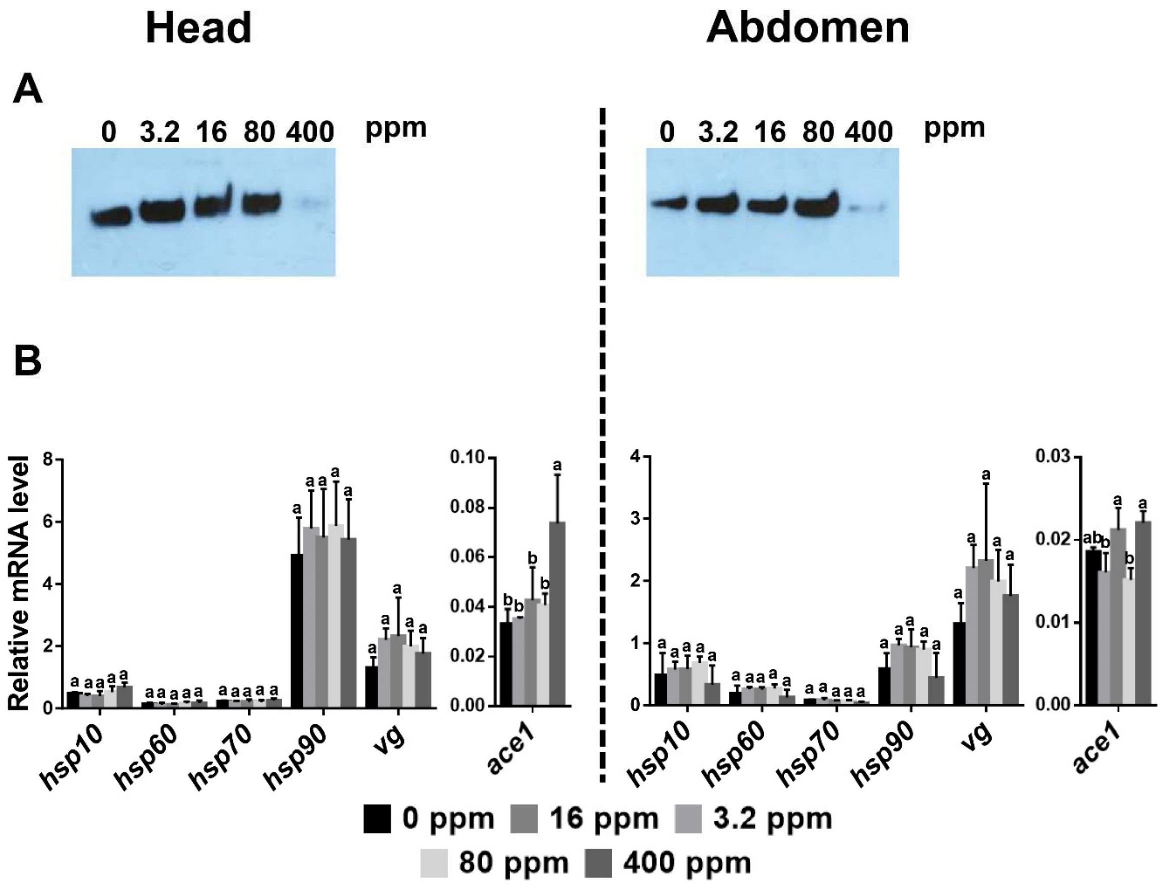


Figure S6. **AmAChE1 expression profile and qPCR results of stress marker genes in the head (A) and abdomen (B) of honey bees with fluvalinate treatment.** Forager bees were incubated in fluvalinate (0, 3.2, 16, 80 and 400 ppm)-coated glass bottles for 14 days. The data was analyzed by one-way ANOVA followed by Tukey's multiple comparison and significant differences were marked with $^{*}$ ($p < .05$), $^{**}$ ($p < .01$) and $^{***}$ ($p < .001$).

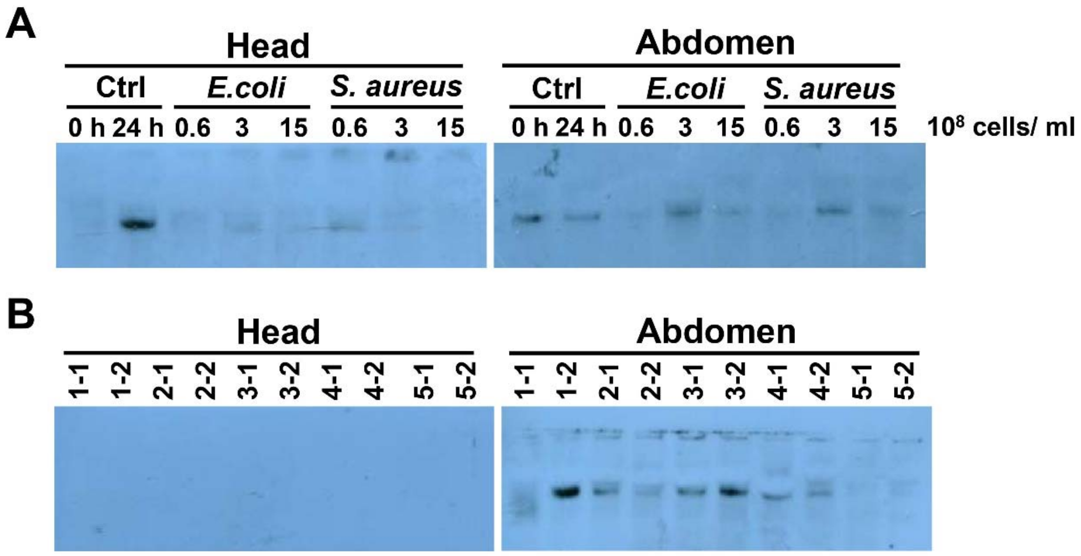


Figure S7. **AmAChE1 expression profile in the head and abdomen of honey bees with bacterial challenge (A) and Varroa mite infestation (B).** LB broth-cultured *Escherichia coli* and *Staphylococcus aureus* were mixed with 10% sucrose solution and provided to nurse bees for 24 h. AmAChE1 expression profile in honey bees' heads and abdomens with Varroa mite infestation (B). 1. Naturally mite-attached nurse bees from infected hive; 2. Naturally mite-unattached nurse bees from infected hive; 3. Naturally mite-unattached nurse bees from clean hive; 4. Naturally mite-unattached nurse bees from clean hive; 5. Artificially mite-attached one from clean hive; Two replicates for every condition and each replicate consisted of 10 units of nurse bees. 1, 2 and 3 samples were directly used and 4 and 5 samples were incubated for 24 h.

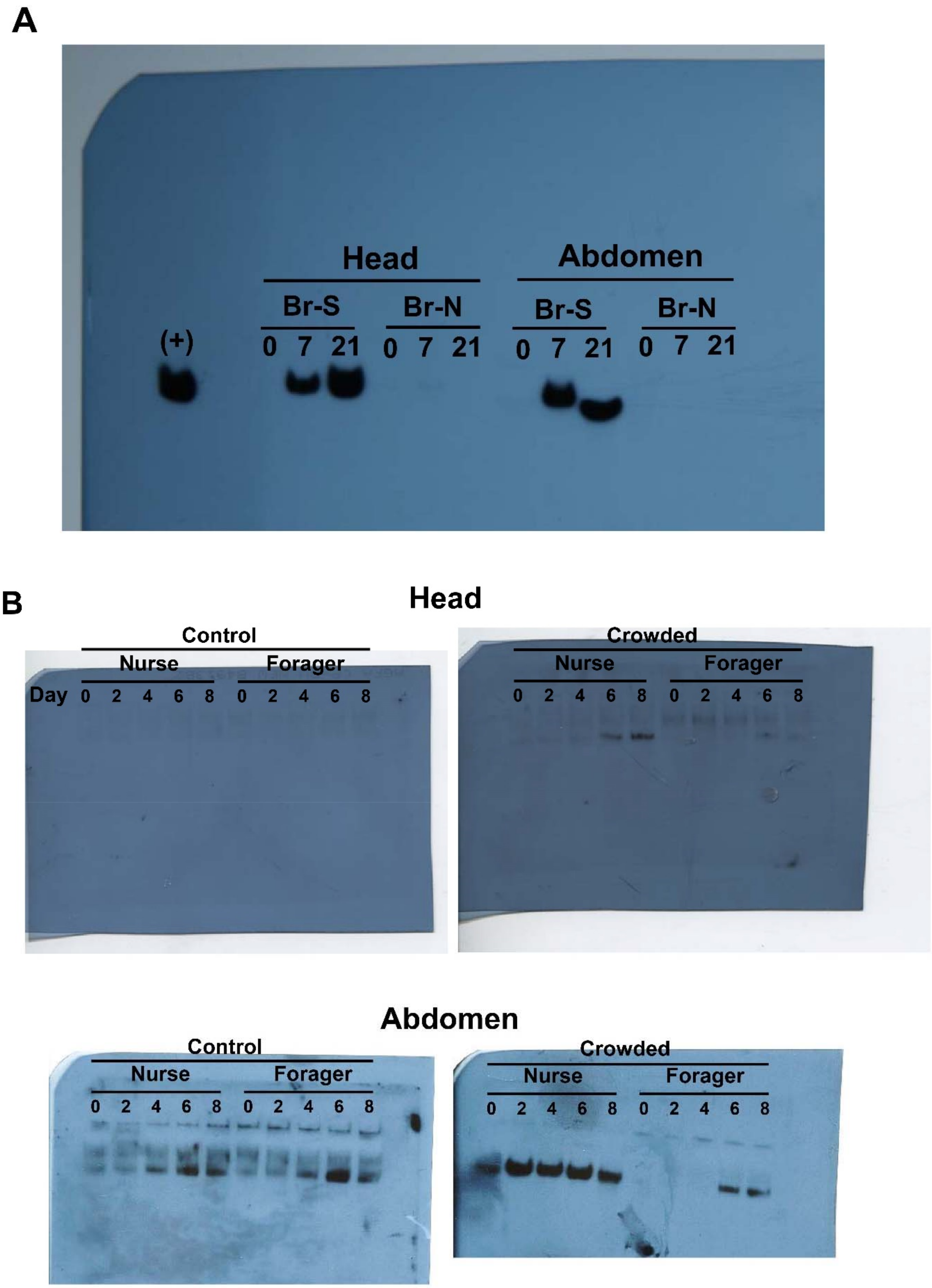
A
Head
Abdomen
Br-S
Br-N
Br-S
Br-N
(+)
0 7 21 0 7 21 0 7 21 0 7 21
B
Head
Control
Crowded
Nurse
Forager
Nurse
Forager
Day 0 2 4 6 8 0 2 4 6 8
0 2 4 6 8 0 2 4 6 8
Abdomen
Control
Crowded
Nurse
Forager
Nurse
Forager
0 2 4 6 8 0 2 4 6 8
0 2 4 6 8 0 2 4 6 8

C

Head

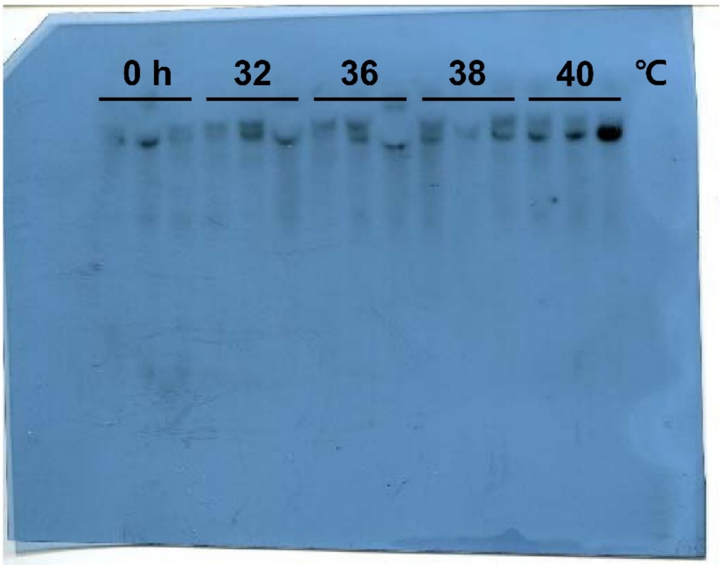


Abdomen

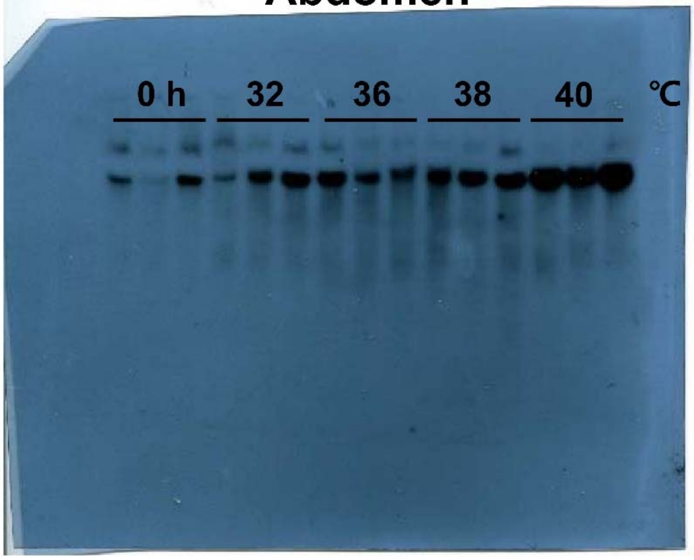

D

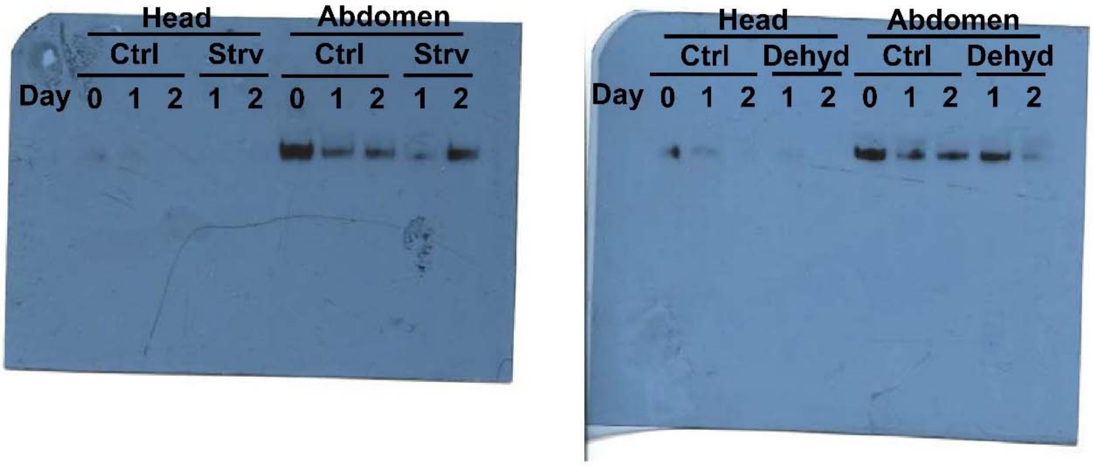
Head
Abdomen
Ctrl
Strv
Ctrl
Strv
Day 0 1 2 1 2 0 1 2 1 2
Head
Abdomen
Ctrl
Dehyd
Ctrl
Dehyd
Day 0 1 2 1 2 0 1 2 1 2

E

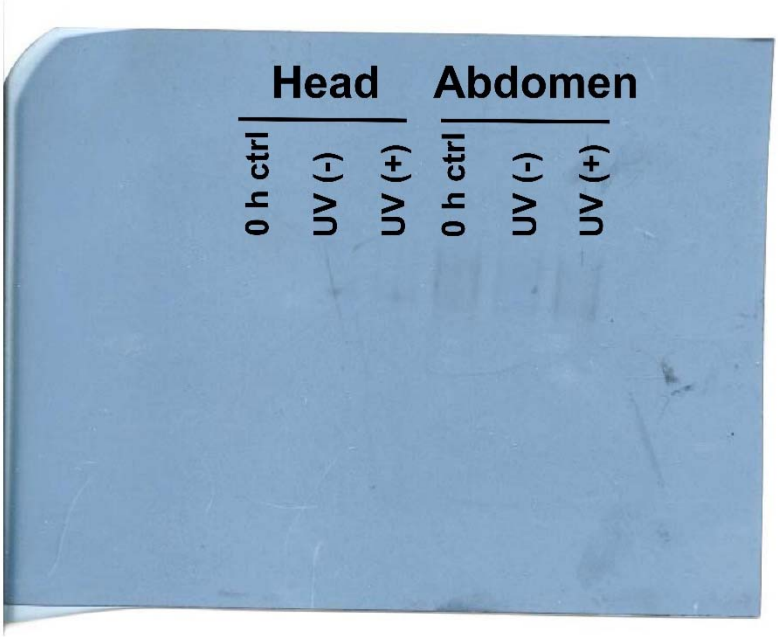
Head
Abdomen
0 h ctrl
UV (-)
UV (+)
0 h ctrl
UV (-)
UV (+)

F

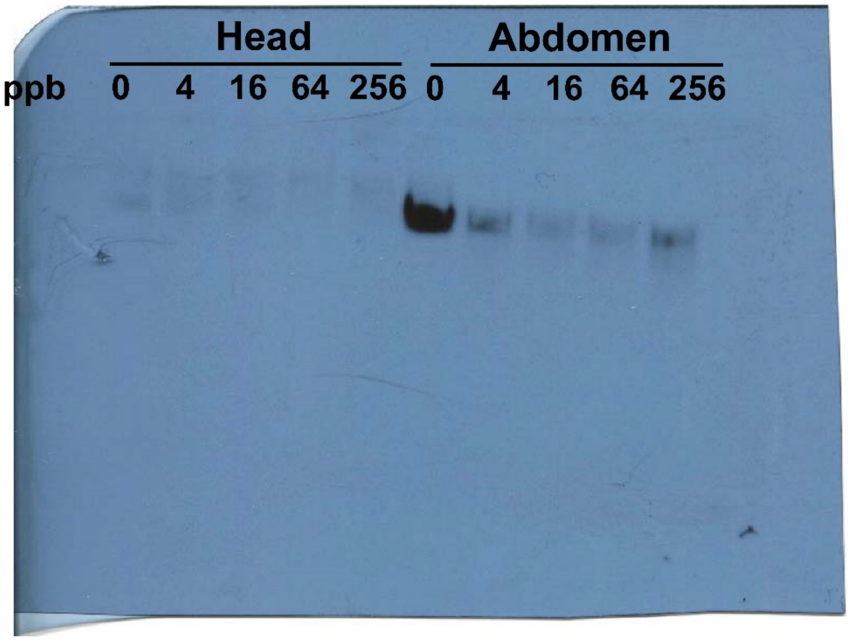
Head
Abdomen
ppb
0 4 16 64 256 0 4 16 64 256

G

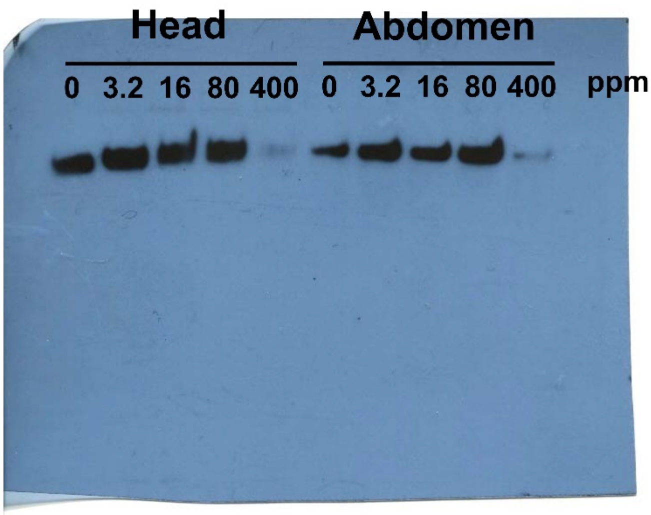
Head
Abdomen
0 3.2 16 80 400 0 3.2 16 80 400 ppm

H

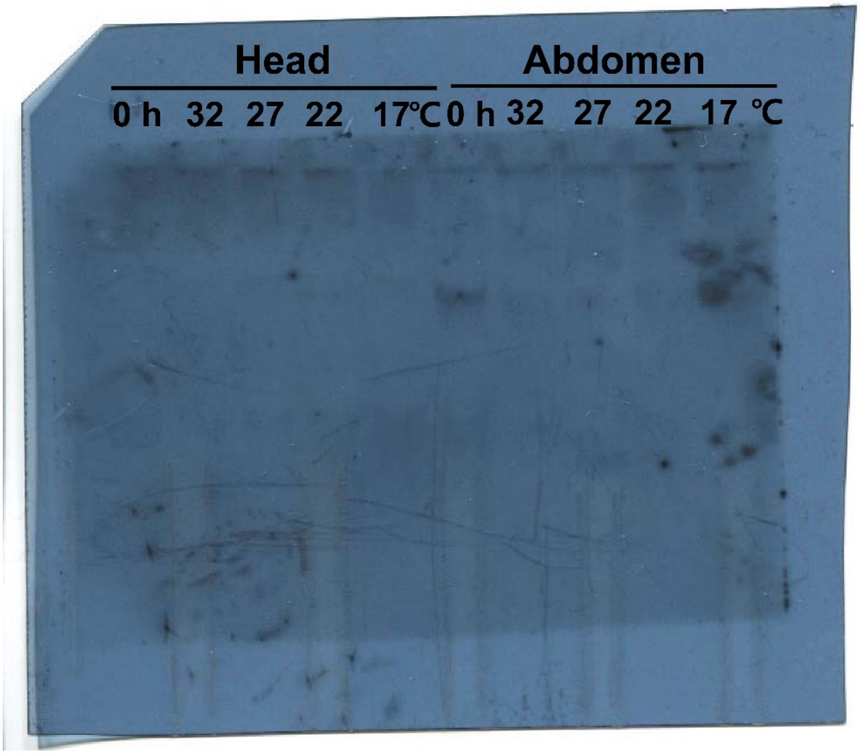
Head
Abdomen
0 h 32 27 22 17℃
0 h 32 27 22 17 ℃

I

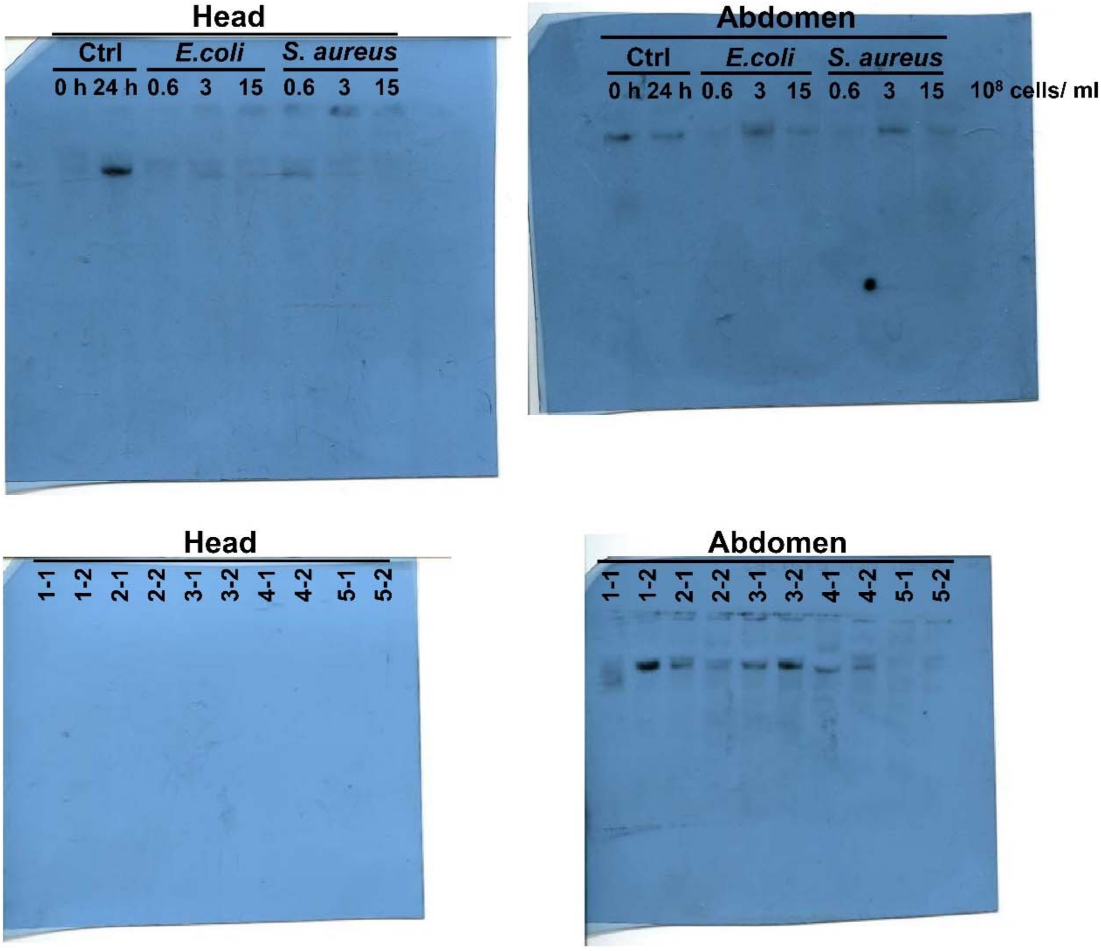
Head
Ctrl
E.coli
S. aureus
0 h 24 h 0.6 3 15 0.6 3 15
Abdomen
Ctrl
E.coli
S. aureus
0 h 24 h 0.6 3 15 0.6 3 15
10$^{8}$ cells/ ml
Head
1-1 1-2 2-1 2-2 3-1 3-2 4-1 4-2 5-1 5-2
Abdomen
1-1 1-2 2-1 2-2 3-1 3-2 4-1 4-2 5-1 5-2

Figure S8. **Full-length blots of Western blotting experiments.** Protein samples extracted from head and abdomen were separated on native-PAGE gel. After PAGE, Western blotting was performed with anti-AChE1 antibody. AmAChE1 expression profile in the head and abdomen of honey bees following brood rearing suppression from Figure 1 (A), crowding from Figure 2 (B), heat shocks treatment from Figure 3 (C), starvation and dehydration from Figure S1 (D), UV-B treatment form Figure S3 (E), imidacloprid treatment from Figure S4 (F), fluvalinate treatment from Figure S5 (G), cold shock treatment from Figure S6 (H) and bacterial challenge and Varroa mite infestation from Figure S7 (I).

Table S1. **Primers list used for qPCR of honey bee genes.**

| Gene name | Primer sequence | Product size | Accession No. |
|---|---|---|---|
| *rps5* | F- AATTATTTGGTCGCTGGAATTG | 114 | XM_006570237.2 |
| | R- TAACGTCCAGCAGAATGTGGTA | | |
| *hsp10* | F- TGTTGTAGCAATTGGACCTGG | 161 | XM_624907.4 |
| | R- TGCCAGTATATCTGACTCACG | | |
| *hsp60* | F- AATGCAGGCGTGGATGCTAG | 134 | XM_392899.6 |
| | R- CCGTACGCACTACTTTCGTTG | | |
| *hsp70* | F- ATCAACCTGGCGTCTTGATTC | 118 | NM_001160072.1 |
| | R- TGAGGTACACCTCTAGGTGC | | |
| *hsp90* | F- GCGACAGAATGAAGTGCCATG | 146 | NM_001160064.1 |
| | R- CAGAATCAAGTGGCAGGCTTG | | |
| *vg* | F- TCAGTAACCAATGCGAGGGC | 137 | NM_001011578.1 |
| | R- CGACATCTCGGTGTCCAATC | | |
| *ace1* | F- GATCGACGGCGCTTTCCTCG | 150 | KU532288.1 |
| | R- GACCCGTCGATGTGGAACAAC | | |
| *ace2* | F- ATATCCGTTCAACAGTGGAACAGT | 105 | KU532289.1 |
| | R- CTCGTTGTTACCGATCAGTATCT | | |
| *CHAT* | F- CCAGATGGTTATGGTTGCGC | 113 | XP_392463.4 |
| | R- TCGAGCGTTTGAGCGAATCG | | |

Table S2. **The number of pumilio-binding sequences in *Amace1* and *Amace2*.**

| Gene name | Region | Pumilio-binding sequence | | |
|---|---|---|---|---|
| | | TGTAAATA | TGTACATA | TGTATATA |
| ***Amace1*** | **Coding region** | - | - | - |
| | **5'-UTR** | 1 | 1 | 4 |
| | **3'-UTR** | - | - | 1 |
| ***Amace2*** | **Coding region** | - | - | - |
| | **5'-UTR** | 2 | 1 | 7 |
| | **3'-UTR** | - | - | - |
